# Aging in Community: Implementation of a Grassroots Program to Support Rural Aging in Place

**DOI:** 10.1093/geroni/igaf122.836

**Published:** 2025-12-31

**Authors:** Heather Fuller, Jane Strommen, Debarati Kole, Bryce Van Vleet, Sharon Query

**Affiliations:** North Dakota State University, Fargo, North Dakota, United States; North Dakota State University, Fargo, North Dakota, United States; North Dakota State University, Fargo, North Dakota, United States; Eastern Mennonite University, Harrisonburg, Virginia, United States; North Dakota State University, Fargo, North Dakota, United States

## Abstract

Though most older adults prefer to age in place, aging in rural communities can pose unique challenges such as geographic distance to resources, lack of social services, and workforce shortages. ND Aging in Community (AIC) is a grassroots program aimed at improving quality of life by increasing community-level support to help older adults age in place in two rural North Dakotan communities. A multidimensional, systematic evaluation was conducted over 4 years to assess the process and impact of program implementation. Across the first two years, three surveys were conducted with community-based steering committee members (N = 20-29) to assess strengths and areas for growth in service development and community engagement. Findings indicated growth and success over time in service implementation, the critical importance of responsiveness to community voices, and persistent challenges related to workforce and geography. During the final three years, annual surveys were conducted with AIC participants (N = 70-99) to assess satisfaction and impact. Findings indicated older adult participants reported improvement over time in confidence living at home, connection to community, and quality of life; additionally, 100% would recommend the program to others. Through an iterative process, evaluation results were used to inform program refinement. In the end, a holistic Aging in Community Framework was developed with a multi-pronged focus on housing, health and wellness, transportation, connection and growth, personal finance and community engagement. This framework provides an adaptable model for replication in other rural communities. Future directions will be discussed, including examining long-term efficacy and sustainability.

